# The Frailty In Residential Sector over Time (FIRST) study: methods and baseline cohort description

**DOI:** 10.1186/s12877-020-01974-1

**Published:** 2021-02-03

**Authors:** Agathe Daria Jadczak, Leonie Robson, Tina Cooper, J. Simon Bell, Renuka Visvanathan, on behalf of the FIRST Study Collaborators, Jonathan Karnon, Hossein Hajiali Afzali, Olga Theou, Solomon Yu, Rachel Milte, Maria Inacio, Julie Ratcliffe, David Wilson, Graeme Tucker, Shin Liau, Mark Q. Thompson

**Affiliations:** 1 National Health and Medical Research Council Centre of Research Excellence Frailty and Healthy Aging, Adelaide, South Australia Australia; 2 Adelaide Geriatrics Training and Research with Aged Care (G-TRAC) Centre, Adelaide Medical School, Faculty of Health and Medical Sciences, University of Adelaide, Adelaide, South Australia Australia; 3 Aged and Extended Care Services, Basil Hetzel Institute for Translational Health Research and The Queen Elizabeth Hospital, Central Adelaide Local Health Network, Adelaide, South Australia Australia; 4 Resthaven Incorporated, Adelaide, South Australia Australia; 5 Centre for Medicine Use and Safety, Faculty of Pharmacy and Pharmaceutical Sciences, Monash University, Melbourne, Australia

**Keywords:** Aged care, Nursing homes, Frailty, Older adults, Aging

## Abstract

**Background:**

The Frailty In Residential Sector over Time (FIRST) Study is a 3-year prospective cohort study investigating the health of residents living in residential aged care services (RACS) in South Australia. The study aims to examine the change in frailty status and associated health outcomes.

**Methods:**

This interim report presents data from March 2019–October 2020. The study setting is 12 RACS from one organisation across metropolitan and rural South Australia involving 1243 residents. All permanent (i.e. respite or transition care program excluded) residents living in the RACS for at least 8 weeks were invited to participate. Residents who were deemed to be medically unstable (e.g. experiencing delirium), have less than 3 months to live, or not fluent in English were excluded. Data collected included frailty status, medical diagnoses, medicines, pain, nutrition, sarcopenia, falls, dementia, anxiety and depression, sleep quality, quality of life, satisfaction with care, activities of daily living, and life space use at baseline and 12-months. Data Linkage will occur over the 3 years from baseline.

**Results:**

A total of 561 permanent residents (mean age 87.69 ± 7.25) were included. The majority of residents were female (*n* = 411, 73.3%) with 95.3% (*n* = 527) being classified as either frail (*n* = 377, 68.2%) or most-frail (*n* = 150, 27.1%) according to the Frailty Index (FI). Most residents were severely impaired in their basic activities of daily living (*n* = 554, 98.8%), and were at-risk of malnutrition (*n* = 305, 55.0%) and at-risk of sarcopenia (*n* = 492, 89.5%). Most residents did not experience pain (*n* = 475, 85.4%), had normal daytime sleepiness (*n* = 385, 69.7%), and low anxiety and depression scores (*n* = 327, 58.9%).

**Conclusion:**

This study provides valuable information on the health and frailty levels of residents living in RACS in South Australia. The results will assist in developing interventions that can help to improve the health and wellbeing of residents in aged care services.

**Trial registration:**

Prospectively registered with the Australian New Zealand Clinical Trials Registry (ACTRN12619000500156).

**Supplementary Information:**

The online version contains supplementary material available at 10.1186/s12877-020-01974-1.

## Background

Frailty is defined as a clinically recognizable state of increased vulnerability to stressors resulting from age-associated decline in reserve and function across multiple physiologic systems, placing the older person at increased risk of poor health outcomes such as hip fracture and physical disability [1]. Frailty also confers a risk of increased use of healthcare resources including hospital admissions, medical consultations and pharmaceuticals [2, 3].

In Australia, research by our team projects that by 2027, there will be approximately 600,000 older (aged 65 years and over) people living with frailty in our community [4]. Those numbers however do not account for older people who are home bound or living in residential aged care services (RACS). RACS in Australia are increasingly responsible for individuals with higher frailty scores and in part this relates to government policies directed at supporting older people age in place in the community [5]. We also know that older people prefer to stay at home longer and so, increasingly people are being assessed as eligible for RACS with higher frailty scores [6].

We have previously described that in one multi-site residential aged care organization, 85.2% of residents were either frail (60.8%) or most-frail (24.4%) when using the Frailty Index (FI) [7], and 73.4% (frail: 37.5%; most-frail: 35.9%) were frail when using the FRAIL-NH, a screening tool specific for residents of RACS [7]. In our previous research, most-frail residents had higher risk of death but lower risk of hospitalization than non-frail residents [3].

Given the high prevalence of frailty, it is vital that we better understand the trajectory and disease burden as well as generate the necessary evidence to guide the development and implementation of best practice frailty management for residents. Research of community-dwelling older adults has confirmed that frailty levels change over time with many individuals remaining stable, some improving, and others deteriorating, but the change in frailty levels over time for those residing in RACS are less known [8]. Identifying the factors contributing to deterioration or improvement could inform the development of intervention and policy strategies to better support re-enablement where appropriate. There is a strong case that resident wellbeing can be improved and this is best illustrated through the seminal work by Professor Singh and colleagues where high-intensity exercise training was feasible in RACS and contributed to improved strength [9].

Now more than ever, longitudinal studies in residential aged care are necessary for the generation of timely evidence to support improvements in quality of care and, therefore, the wellbeing of residents. There is much interest in improving the quality of aged care services. In Australia, for example, the Royal Commission into Aged Care Quality and Safety currently underway, is an independent investigation with the ultimate goal of providing recommendations to government about changes necessary to improve the quality of aged care services [10].

The aim of the Frailty In Residential Sector over Time (FIRST) Study is to better understand 1) the change in resident frailty over time, 2) the costs and consequences of frailty; and 3) the factors associated with change that could be amenable to intervention. This initial paper describes the study design and the characteristics of the baseline study cohort.

## Methods

### Study design

This is a 3-year prospective cohort study conducted in South Australia, Australia, in collaboration with Resthaven Incorporated (Inc.), a South Australian not for profit aged care provider with 12 services and 1243 residents, representing 6.8% of total residential aged care residents (*n* = 18,375) in South Australia [11]. Data was collected at baseline (March–October 2019) and at 12-months follow up (March–October 2020) with data linkage to occur at 12, 24 and 36 months from baseline.

In Australia, the Aged Care Assessment Program determines eligibility for entry to RACS as one national system. Australian RACS provide supported accommodation for older people with care needs that can no longer be met in their own homes, and are synonymous with ‘nursing homes’ in other countries [12]. The Australian government allocates residential aged care licenses to organizations through the Aged Care Approvals Round. As of the 30th of June 2019 there were 873 organizations (55% not for profits) supporting 213,397 aged care places through 2717 services [11].

Seven of the 12 services were located in the metropolitan area, two in the outer metropolitan area, and three services were regional. The service sizes ranged from 74 to 153 beds, with the majority of services (7 services) sized between 80 to 100 beds. For many years now, there has been an interest in the development of Teaching and Research with Aged Care Services (or ‘Teaching Nursing Homes’) as a means of improving the quality of aged care services [13], and this project is the outcome of such a partnership.

### Recruitment and participants

All permanent (i.e. respite or transition care program excluded) residents of the RACS who were living in the service for at least 8 weeks were invited to participate in the study. Those residents deemed by clinical staff to be medically unstable (e.g. experiencing delirium) or estimated to have less than 3 months to live were excluded. Residents where clinical staff deemed participation to be inappropriate, and those not fluent in English or who had difficulty understanding English, were also excluded.

Study nurses hired by Resthaven Inc. were registered nurses and trained by the research team to obtain informed consent and undertake the data collection across the services. Redcap (Research Electronic Data Capture), a secure electronic web database, was used to collect data across the services and upload any relevant documents. Study nurses were equipped with tablets connected to the service’s wireless network.

Clinical staff at each Resthaven service advised the study nurses prior to data collection on which residents were able to provide informed consent. This was based on residents’ cognitive status using the Psychogeriatric Assessment Scale (PAS) [14] (cut-off score ≥16), as well as resident’s progress and case reports. If clinical staff deemed a resident not to be able to give consent, their substitute decision maker (SDM) was contacted. Residents (or SDM) were provided with a study flyer, a participant information sheet, and a study consent form. Residents and SDM had time to consider the study prior to consent and could withdraw at any time. A separate DHS consent form to access Medicare Benefits Schedule (MBS) and Pharmaceutical Benefits Scheme (PBS) data has been obtained from the residents (or SDM) at 12 months follow-up.

### Data collection and assessments

**Fig. 1:**
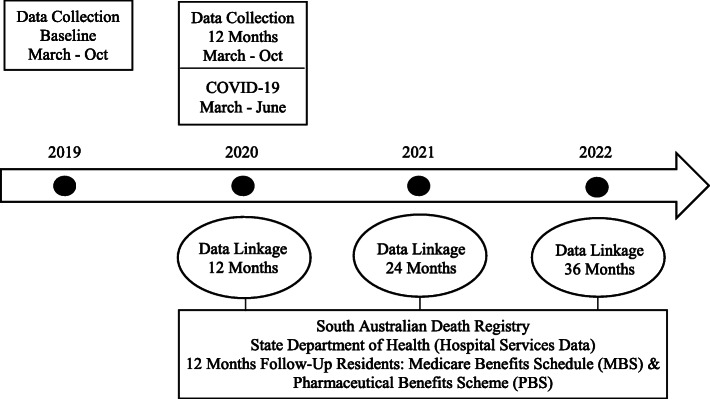
FIRST Study Timeline

Data were collected from residents and site-registered nurses at baseline (March–October 2019), and the study is currently in the midst of data collection at 12 months (March–October 2020) with some changes to the methods to accommodate for the COVID-19 pandemic in 2020 (Fig. 1). The data collection booklet is available as a supplementary file (Additional file 1).

Data were collected from residents’ records, observations, physical assessments and questionnaires conducted with the residents only or with the site-registered nurse at the respective aged care service who was required to have known the resident for at least the past 2 weeks (Table 1). Baseline interviews with the site-registered nurse were conducted within 5 days of initial interview date.

**Table 1:** FIRST Study Assessments

Source	FIRST Study Assessment	Baseline Mar-Oct 2019	12 Months	
			Mar-Jun 2020	Jun-Oct 2020
Residents’ Records	Socio-Demographic Information	✓	✓	✓
	Medical Health History	✓	✓	✓
	Medication Chart	✓	✓	✓
	Falls Report (over 12 months)	✓	✓	✓
	MNA-SF (if ≤3 months)	✓	✓	✓
	KATZ ADL	✓	✓	✓
Resident or Site RN	QoL-AD	✓	Site RN Only	Site RN Only
	Epworth Sleepiness Scale	✓	Site RN Only	Site RN Only
	PHQ-4 Anxiety and Depression	✓	Site RN Only	Site RN Only
Site RN	NHLSD	✓	✓	✓
	DSRS	✓	✓	✓
	MNA-SF (if >3 months)	✓	✓	✓
	SARC-F	✓	Not Done^a^	✓
Resident	CCI-6D	✓	Not Done^a^	✓
	PWI	✓	Not Done^a^	✓
Physical Assessment	Grip Strength	✓	Not Done^a^	✓
Observations	POSS	✓	Not Done^a^	✓
	PAINAD	✓	Not Done^a^	✓

^a^Due to COVID-19 restrictions/modifications, SARC-F omitted due to missing grip strength assessment; *MNA-SF* Mini Nutritional Assessment Short Form, *KATZ ADL* Katz Activities of Daily Living Scale, *QoL-AD* Quality of Life in Alzheimer’s Disease Scale, *PHQ-4* Patient Health Questionnaire-4, *NHLSD* Nursing Home Life-Space Diameter, *DSRS* Dementia Severity Rating Scale, *POSS* Pasero Opioid Induced Sedation Scale, *PAINAD* Pain Assessment in Advanced Dementia, *CCI-6D* Consumer Choice Index 6 Dimension, *PWI* Personal Wellbeing Index, *RN* Registered Nurse

#### Residents’ records

Socio-demographic information (service, gender, date of birth, primary language, Medicare number, and country of birth), medical health history, weight over the past 3 months, height, medicines, number of falls (over the past 12 months), and Activities of Daily Living (ADL) were obtained from residents’ service records on the day of interview.

Medical health history included myocardial infarction, congestive cardiac failure, peripheral vascular disease, cerebrovascular disease (or stroke), atrial fibrillation, hypertension, diabetes, history of delirium, Parkinson’s Disease, dementia, depression, anxiety, chronic obstructive pulmonary disease, arthritis, osteoporosis, hip fracture, other fractures, gout, pressure injuries, leg ulcers, ulcer disease, connective tissue disease, urinary incontinence, faecal incontinence, skin cancers, other cancers, any tumor, hearing impairment, dry eyes and glaucoma. Insomnia was recorded if present in residents’ medical health history or if the total score of the Epworth Sleepiness Scale (ESS) [15] was ≥11. Falls were present if residents had ≥1 fall over the past 12 months.

Medication charts included prescription and non-prescription medications administered regularly and as required (PRN), which were classified using the Anatomical Therapeutic Chemical (ATC) classification system. Polypharmacy was defined as having ≥9 regular medications [16]. This included oral, inhaled and transdermal formulations but excluded dietary supplements, alcohol, short-term medicines (i.e. antibiotics) PRN medicines, topical lotions, creams and ointments used in wound care (non-wound care related lotions, creams and ointments were included). Different strength products of the same medication were counted as one medication. Falls were defined as slipping, tripping, rolling and sliding resulting in the resident coming to rest inadvertently on the ground, floor or other lower level [17].

ADLs were assessed using the KATZ ADL Scale [18] including bathing, dressing, toileting, transferring, continence, and feeding. The study nurse competed the KATZ ADL Scale based on residents’ records and care plans. Any physical assistance needed, including supervision, was classified as not independent (score 0). Only residents who were deemed to be independent in their service records received a score of 1 (independent). A total score of 6 indicates fully independent, 4 indicates moderate independent, and 2 or less indicates not independent.

#### Observations and physical assessment

The study nurse observed each resident for 5 min before completing the Pain Assessment in Advanced Dementia (PAINAD) Scale [19] and the Pasero Opioid-induced Sedation Scale (POSS) [20]. The PAINAD was used to determine residents’ level of pain and includes five domains related to breathing, negative vocalization, facial expressions, body language, and consolability. The scores range from 0 to 10 with higher scores indicating more severe pain [19]. The POSS was used to determine residents’ daytime sedation. A POSS score of ≥2 indicates an acceptable level of sedation whereas a score of 3 or 4 indicates over-sedation [20].

Grip strength was assessed using a Martin-Vigorimeter (KLS Martin Group, Tuttlingen, Germany). Up to three attempts with residents’ dominant hand were recorded and the mean was used to answer the strength related question in the SARC-F questionnaire [21]. A grip strength of 0.7–1.3 bar (based on KLS Martin Group standard value for healthy adults) was considered as ‘no difficulty in lifting and carrying 10 pounds’ in the SARC-F questionnaire. A result of ≤0.69 bar was considered as ‘some difficulty’, and if the resident was unable to perform the grip strength test, the answer to the strength related SARC-F question was ‘a lot of difficulty or unable’.

#### Questionnaires with resident

The Consumer Choice Index Six Dimension (CCI-6D) [22] and the Personal Wellbeing Index (PWI) [23] were conducted with the resident only. The CCI-6D is a new instrument designed specifically to evaluate the quality of care received in long-term care from a consumer perspective. It includes a scoring algorithm developed based on the preferences of people living in residential aged care with scores ranging from 0 (poor quality care) to 1 (high quality care) [24]. The PWI was used to assess resident’s satisfaction with seven quality of life domains including standard of living, health, achieving in life, relationships, safety, community-connectedness, and future security. Scores range from 0 to 70 with higher scores indicating higher satisfaction [23].

#### Questionnaires with resident or site-registered nurse

The Quality of Life in Alzheimer Disease (QoL-AD) Scale [25], the ESS [15], one question from a sleep quality questionnaire regarding napping frequency [26], and the Patient Health Questionnaire-4 (PHQ-4) [27] were completed by the resident where possible, or else by the site-registered nurse at the respective aged care service.

Quality of life was assessed using the QoL-AD [25]. The original 13-item version was administered at baseline and at 12 months follow-up, while the 15-item version developed for use in RACS, was introduced at 12 months follow-up only. The 15-item version for RACS does not include questions related to money and marriage, but has four additional items related to (1) people who work here, (2) ability to take care of oneself, (3) ability to live with others, and (4) ability to make choices in one’s life [28]. Further, the wording of one question has been changed (‘ability to do chores’ was changed to ‘ability to keep busy’). Scores range from 13 to 52 (13-item version) or 15 to 60 (15-item version) with higher scores indicating better quality of life. In cases where one or two items were missing, those were imputed with the mean score of the remaining items to calculate the total score. If more items were missing, the QoL-AD was discarded [25].

The ESS [15] and the question from a sleep quality questionnaire regarding napping frequency [26] were used to assess residents’ daytime sleepiness. The ESS score (the sum of 8 items) ranges from 0 to 24. The higher the ESS score, the higher is the daytime sleepiness [15].

The PHQ-4 [27] is a 4-item questionnaire and was used to determine residents’ risk of depression and anxiety. Scores range from 0 to 12 and are rated as normal (0–2), mild (3–5), moderate (6–8), and severe (9–12). A total score of ≥3 for the first 2 questions suggests anxiety, and a total score of ≥3 for the last 2 questions suggests depression.

#### Questionnaires with site-registered nurse

For the remaining questionnaires, the study nurse interviewed the site-registered nurse on the day of data collection or else within 5 days of interview date. Where a face-to-face interview was not possible, the study nurse left the questionnaires with the site-registered nurse to be completed within 5 days.

Residents’ risk of sarcopenia was assessed using the SARC-F [21]. The SARC-F includes 5 questions regarding strength, walking, chair rise, stairs and falls. Strength was prepopulated from the grip strength assessment, and number of falls was prepopulated from residents’ records. A total score of ≥4 indicates a risk of sarcopenia.

The Nursing Home Life Space Diameter (NHLSD) [29] was used to investigate residents’ degree and frequency of mobility. The NHLSD includes four diameters: within the room, outside the room but within the unit (or wing), outside the unit but within the service, and outside the service. The scores range from 0 to 50 with higher scores indicating a greater use of life space.

The Dementia Severity Rating Scale (DSRS) [30] is a 12-item questionnaire and was used to assess the severity of residents’ dementia. Scores range from 0 to 54 and can be rated as no dementia (0–11), mild dementia (12–18), moderate dementia (19–36) and severe dementia (37–54) [31].

The Mini Nutritional Assessment Short Form (MNA-SF) [32] was used to assess residents’ nutritional status. The MNA-SF is conducted regularly as a part of routine care in the RACS and where the MNA-SF had been completed in the past 3 months, questions relating to food intake and psychological stress were obtained from residents’ records, otherwise from the site-registered nurse. Questions relating to weight loss and Body Mass Index (BMI) were prepopulated from the weight and height data obtained from the records. The mobility related question was prepopulated from answers to DSRS question 12 (bed or chair bound: DSRS 12 score 5–6; able to get out of bed/chair but does not go out: DSRS 12 score 3–4; goes out: DSRS 12 score 0–2). The question related to neuropsychological problems was prepopulated from the DSRS total score and the sum of PHQ-4 question 3 and 4 (severe dementia or depression: total DSRS ≥19 or PHQ-4 question 3 & 4 is ≥3; mild dementia: total DSRS 12–18; no psychological problems: total DSRS 0–11 and PHQ-4 question 3 & 4  is <3). MNA-SF scores range from 1 to 14 and are rated as normal (12–14), at-risk of malnutrition (9–11), and malnourished (0–7) [32].

#### Frailty assessments

Two frailty measures were used to assess residents’ frailty status: the Frailty Index (FI), which follows an accumulation of deficit approach [33], and the FRAIL-NH, a frailty screening tool specifically developed for RACS [34].

A 60-item FI was constructed from baseline descriptors and co-variates following a standard methodology [35]. The variables used to construct the FI were similar to those used in our previous study [7]. The FI variables used in this study included 22 comorbidities, 3 items from KATZ ADL [36], 9 items from the 13-item QoL-AD [25], 4 items from MNA-SF [32], 1 item from POSS [20], 6 items from ESS [15], 1 item from the sleep quality questionnaire [26], 2 items from PAINAD [19], and 12 items from DSRS [30] (Additional file 2). Each variable was coded to provide a score between 0 (absence of deficit) and 1 (representing full expression of deficit). The FI score, representing the proportion of deficits present for each resident, was calculated by dividing the sum of recorded deficits by the total number of FI variables available. A FI score was not reported for those residents who were missing responses to > 20% of FI variables. Residents were classified into the following frailty categories based on their proportion of deficits present: non-frail (0 to ≤0.1), vulnerable (>0.1 to ≤0.21), frail (>0.21 to <0.45), and most-frail (≥0.45 or more) [37].

The FRAIL-NH [34] is a frailty screening tool and includes seven items regarding energy, transferring, mobility, continence, weight loss, nutrition and dressing. The FRAIL-NH was constructed from variables drawn from data collected for this study including PHQ-4 question 3 and 4 for energy, MNA-SF question 2 for weight loss, SARC-F question 3 for transferring, and DSRS question 9, 10, 11 and 12 for dressing, feeding, continence and mobility, respectively (Additional file 3). FRAIL-NH scores range from 0 (best) to 14 (worst).

### COVID-19 modifications

At the commencement of 12 months follow-up, face-to-face data collection was ceased for five RACS when visitation to all 12 RACS was restricted to all visitors with the exception of those visiting a resident at end of life, supporting a resident’s attendance at an urgent medical appointment, or on compassionate terms as approved by the RACS Manager. Those restrictions were in place between the 25th March 2020 and 3rd May 2020 with visitor restrictions gradually easing throughout May 2020. Face-to-face data collection resumed from the 22nd June 2020 and was fully in place by the 26th June 2020 across all RACS (Table 1). All questionnaires, observations and assessments able to only be completed by the residents were ceased from the 25th March until the 22nd June 2020 including the PAINAD, POSS, CCI-6D, PWI and the grip strength assessment (and so the SARC-F). During this time, the site-registered nurse completed the Qol-AD, ESS, and PHQ-4, and this continued beyond the 22nd June 2020 to reduce resident exposure time to the study nurse. No modifications were made in relation to the collection of the NHLSD, DSRS and MNA-SF. The questionnaires were either posted out to the site-registered nurse at the respective RACS to complete, or the site-registered nurse visited Resthaven Inc. Headquarters to complete the questionnaires within +/− 10 days of initial baseline interview date. To date, no residents have tested positive to COVID-19 across any of the 12 RACS for this organization.

### Data linkage

Data linkage will occur via SA NT DataLink at 12, 24 and 36 months from baseline. Data requested from Medicare will provide information on costs associated with out of hospital services (e.g., general practitioner (GP) visits), and pharmaceuticals. Data on hospital services and mortality will be collected from the State Department of Health Integrated South Australian Activity Collection (ISAAC) and State Death Registry, respectively. Following collection, the data from alternative sources will be matched to create an individual record for each resident.

### Statistical analysis

Pearson correlation coefficient investigated the association between the FI and the FRAIL-NH. Receiver operating characteristic (ROC) curves were used to establish cut points for the FRAIL-NH by examining the FRAIL-NH score that maximized sensitivity and specificity in predicting frailty and severe frailty based on the FI. Chi-square test was used to examine the association between service size, service rurality and frailty group (vulnerable/frail and most-frail). Analyses were conducted using SPSS version 25 (IBM Corporation. Armonk, NY). The level of statistical significance was set at *p* < 0.05. Descriptive data is presented as mean and standard deviation (SD).

## Results

### Recruitment

**Fig. 2:**
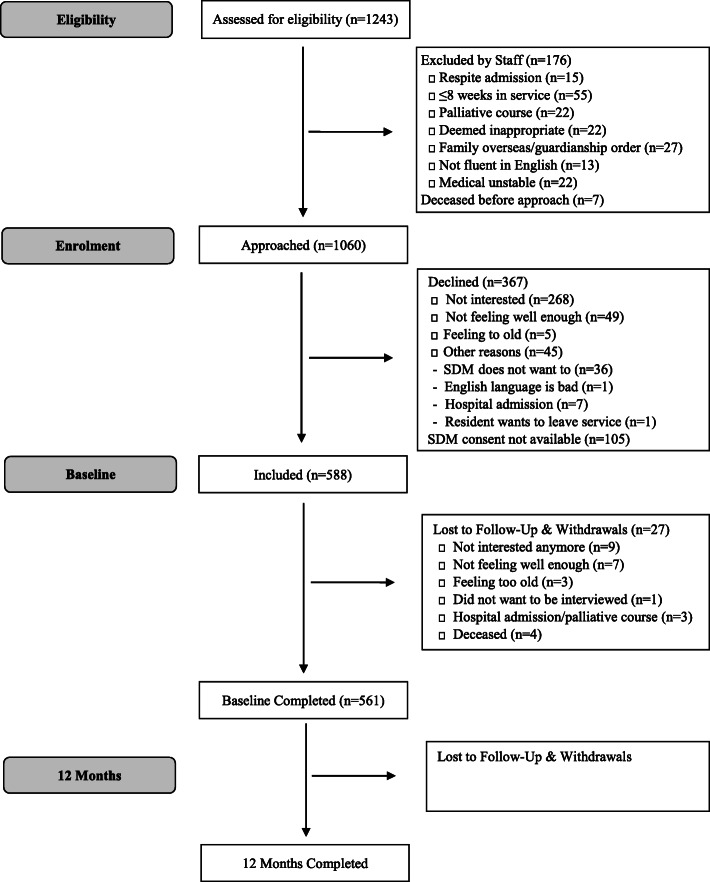
FIRST Study Consort Flow Diagram

Out of 1243 residents who were residing in the 12 services, 1060 (85.3%) residents were eligible for the study (Fig. 2). A total of 472 (45.2%) of those eligible either declined (*n* = 367, 34.6%) or their SDM was not available (*n* = 105, 9.9%) resulting in 588 residents (55.5%) being included in the study and 561 (52.9%) completed baseline data collection. There were 26 withdrawals and 1 person did not want to be interviewed (Fig. 2).

### Participants’ frailty levels

Based on the 60-item FI, none of the residents were non-frail, 4.7% (*n* = 26) were vulnerable, and 95.3% (*n* = 527) of residents were either frail (*n* = 377, 68.2%) or most-frail (*n* = 150, 27.1%).

### Participants’ baseline health characteristics

At baseline (Table 2), the mean age was 87.69 ± 7.25 years with the majority of residents female (*n* = 411, 73.3%) and with dementia as per the DSRS score of ≥11 (*n* = 422, 75.9%). Most of the residents were dependent with their ADLs (*n* = 554, 98.8%), at-risk of malnutrition (*n* = 305, 55.0%) and at-risk of sarcopenia (*n* = 492, 89.5%). The majority of residents had experienced ≥1 fall in the past 12 months (*n* = 340, 60.6%). Most residents were not in pain (*n* = 475, 85.4%) with the majority exhibiting normal daytime sleepiness (*n* = 385, 69.7%) and low anxiety and depression scores (*n* = 327, 58.9%). Residents’ QoL scores ranged from 15 to 52 with a mean score of 34.03 ± 6.66. Their perception of the quality of care ranged from 0.10 to 1.00 with a mean score of 0.77 ± 0.20, and residents’ satisfaction with life ranged from 10 to 70 with a mean score of 55.84 ± 10.05.

**Table 2:** Participant Baseline Characteristics

Characteristic	Total
Total	561
Age, mean (SD)	87.69 (7.25)
Female, n (%)	411 (73.3)
Body Mass Index, mean (SD)	26.62 (6.16)
Number Falls^a^, mean (SD)	3.53 (7.89)
0 Falls, n (%)	221 (39.4)
≥1 Fall, n (%)	340 (60.6)
Comorbidities	
Myocardial Infarction, n (%)	158 (28.3)
Stroke, n (%)	165 (29.6)
Chronic Obstructive Pulmonary Disease, n (%)	133 (23.8)
Dementia, n (%)	207 (37.1)
Diabetes, n (%)	133 (23.8)
Polypharmacy (≥9 Med), n (%)	359 (64.0)
Pain Assessment in Advanced Dementia (PAINAD), mean (SD)	0.14 (0.62)
No Pain (0), n (%)	475 (85.4)
Mild Pain (1–3), n (%)	74 (13.3)
Moderate Pain (4–6), n (%)	7 (1.3)
Epworth Sleepiness Scale (ESS), mean (SD)	7.86 (5.94)
Normal (0–10), n (%)	385 (69.7)
Mild Excessive (11–12), n (%)	50 (9.1)
Moderate/Severe (13–24), n (%)	117 (21.2)
Pasero Opioid Induced Sedation Scale (POSS), mean (SD)	1.10 (0.54)
Katz Activities of Daily Living (Katz ADL), mean (SD)	0.32 (0.63)
Fully Independent (5–6), n (%)	0 (0.0)
Moderately Independent (3–4), n (%)	7 (1.2)
Not Independent (0–2), n (%)	554 (98.8)
Nursing Home Life Space Diameter (NHLSD), mean (SD)	27.86 (10.12)
Frailty Index 60-Item, mean (SD)	0.37 (0.11)
Screening Tool Sarcopenia (SARC-F), mean (SD)	6.46 (2.1)
At Risk (≥4), n (%)	492 (89.5)
Mini Nutritional Assessment Short Form (MNA-SF), mean (SD)	10.15 (2.43)
Normal (12–14), n (%)	172 (31.0)
At Risk (8–11), n (%)	305 (55.0)
Malnourished (0–7), n (%)	78 (14.1)
Dementia Severity Rating Scale (DSRS), mean (SD)	23.59 (14.11)
No Dementia (0–11), n (%)	134 (24.1)
Dementia (>11), n (%)	422 (75.9)
Mild (12–18), n (%)	96 (17.3)
Moderate (19–36), n (%)	198 (35.6)
Severe (37–54), n (%)	128 (23.0)
Patient Health Questionnaire-4 (PHQ-4), mean (SD)	2.88 (3.22)
Normal (0–2), n (%)	327 (58.9)
Mild (3–5), n (%)	122 (22.0)
Moderate/Severe (6–12), n (%)	106 (19.1)
Quality of Life Alzheimer Disease (QoL-AD) 13-Item, mean (SD)	34.03 (6.66)
Consumer Choice Index-6 Dimension (CCI-6D), mean (SD)	0.77 (0.20)
Personal Wellbeing Index (PWI) 7-Item, mean (SD)	55.84 (10.05)

^a^Falls include slipping, tripping, rolling or sliding to a lower level over past 12 months; *SD* Standard Deviation

### FRAIL-NH cut points

**Fig. 3:**
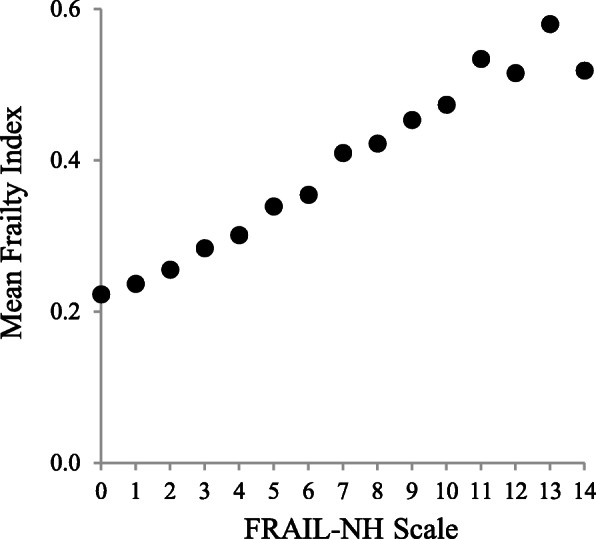
60-Item Frailty Index Mean Score for each point on the FRAIL-NH Scale

The FRAIL-NH was significantly correlated with the FI (r = 0.77, *p* < 0.001) (Fig. 3). ROC analysis of the FRAIL-NH against the FI showed that the area under the curve was 0.89 (95% confidence interval (CI) = 0.85–0.94) for frail residents and 0.88 (95% confidence interval (CI) = 0.86–0.91) for most-frail residents. The FRAIL-NH scores with the highest sensitivity and specificity in classifying frailty and severe frailty based on the 60-item FI were 3 (80.7% sensitivity, 84.0% specificity) and 7 (81.6% sensitivity, 79.1% specificity), respectively, resulting in the following categories for the FRAIL-NH: non frail (0–2), frail (3–6), most-frail (7–14). Based on these FRAIL-NH cut-offs, 12.3% (*n* = 68) were considered as non-frail, and 87.7% (*n* = 484) were either frail (*n* = 232, 42.0%) or most-frail (*n* = 252, 45.7%).

### Service size and rurality

The majority of residents (92.9%) resided in larger services of > 80 beds and were located in the metropolitan area (67.1%). No associations were found between frailty levels and service size (*p* = 0.139) or service rurality (*p* = 0.116) (Table 3).

**Table 3:** Residential Aged Care Service Characteristics and Frailty Levels

Characteristic	Total n	Vulnerable/Frail^**a**^ n (%)	Most-Frail^**a**^ n (%)	***P***-value
Service Size				
60–80 beds	39	32 (82.1)	7 (17.9)	0.139
81–100 beds	271	203 (74.9)	68 (25.1)	
≥101 beds	243	168 (69.1)	75 (30.9)	
Service Rurality				
Metropolitan Area	371	267 (72.0)	104 (28.0)	0.116
Outer Metropolitan	76	51 (67.1)	25 (32.9)	
Regional	106	85 (80.2)	21 (19.8)	

^a^Frailty based on 60-item Frailty Index available for 553 residents

## Discussion

The FIRST Study baseline cohort provides valuable information on the frailty levels and health characteristics of residents living in RACS in South Australia. Research indicates that aged care organizations are increasingly managing very frail residents (5) and this is confirmed when the findings of this research are compared to our previous research (conducted April–August 2014) with the same organization [7]. Frailty levels determined by the FI were higher in this study with 95.3% of residents being considered to be either frail (FI >0.21 to <0.45; 68.2%) or most-frail (FI ≥0.45; 27.1%) compared to 85.2% (frail: 60.8% or most-frail: 24.4%) 5 years ago [7]. These findings are consistent with the trends of increasing frailty noted in relation to those approved for and then entering RACS over a period of 10 years (2006–2015) where there has been an increase in the proportion of frail (FI ≥0.2) people approved from 69 to 87.2% and most-frail (FI ≥0.4) from 0.6 to 3.3% [6]. It is possible that people are being approved for RACS only at a higher frailty levels given the increased effort at helping older people stay at home longer through community aged care services (i.e. home care packages). In the 2017–2018 financial year, 116,800 people were supported by home care packages, an increase from the 97,200 that were the numbers supported the previous financial year [38]. Australia when compared to the Organisation for Economic Co-operation and Development (OECD) average of 42.8 places per 1000 population aged 65 years and over remains well placed with 51 residential aged care places per 1000 population aged 65 years and over [39]. Supporting a need to shift towards community aged care services, we have previously reported that older people continue to wait for long periods for home care packages and this is associated with increased risk of mortality and placement into RACS [40]. Frailty levels in this population are likely to be higher given that we excluded from our study residents who were medically unstable (*n* = 22, 1.8%) or receiving palliative care (n = 22, 1.8%). It is commonly assumed that as people age in RACS, they are likely to become frailer [6] but this study will allow us to clarify if this is the only trajectory or if there are individuals that improve over time as has been seen in community-dwelling older adults [8].

Where frailty exists, geriatric syndromes are common and should be screened for. In having to deal with the increasing burden of frailty, RACS are increasingly being called upon to better manage geriatric syndromes such as falls, polypharmacy, under-nutrition and dementia. In this study, 75.6% of residents had dementia with moderate to severe dementia being recorded for 58.6% of the residents. Additionally, when compared to our previous research in the same organization (conducted in 2014), the current mean DSRS score was higher (mean ± SD: 23.6 ± 14.1 vs. 19.1 ± 16.9) [41]. The finding that three quarters of residents had dementia is in line with findings elsewhere. For example, a Swedish study reported that the prevalence of dementia was as high as 85% in 2010/2011 [42].

Under-recognition of geriatric syndromes remains common with previous studies reporting that when discharging to RACS from hospitals, geriatric syndromes are typically not recorded in discharge summaries. In fact, in Australian research of people being assessed for eligibility and then accessing RACS, the dementia prevalence recorded was 11% in 2015 [6], well below the almost 60% of moderate to severe dementia reported in this study. When medical record alone were considered, the prevalence of dementia in our study was lower at 37.1% and the need to confirm the dementia prevalence using more than one source was recently confirmed in another Australian study where a higher prevalence of 57.8% was noted when a combination of the aged care funding instrument (ACFI) and electronic health records (EHR) was used compared to the ACFI (47.5%) or EHR (48.9%) alone [43]. There can therefore be no doubt that now more than ever, moving forward, educational and training programs for staff working in RACS, including visiting GPs, should deliver on skills that support earlier recognition and management of geriatrics syndromes [44].

The Healthy Quality and Safety Commission of New Zealand have developed frailty care guides for aged care whereby the FRAIL-NH is recommended for use at admission, routine review and 2–4 weeks after an acute event to support stabilization, care planning and timely intervention [45]. We developed a FRAIL-NH modified from the original version [34] with questions derived from the dementia (DSRS), anxiety/depression (PHQ4), nutrition (MNA-SF) and sarcopenia (SARC-F) questionnaires. Because the questions used were different to those in our previous research [7], we developed new cut-offs. Our cut-off for non-frail was 2, which was lower than that previously seen in our research (i.e. 3) [7], and very much lower than that proposed for the original scale (i.e. 5) [34]. The reason for difference relates to the differing cut-off used to define frailty with frailty defined in the original paper as a FI ≥0.3, whilst it was defined as FI >0.21 in this research. Therefore, when comparing studies using the FRAIL-NH, it is important to note that the use of different questions as well as different cut-offs influences reported prevalence and outcomes.

Similar to that reported for Australia, approximately 67% of residents were residing in metropolitan region [6]. In this study service characteristics (i.e. size and rurality) were not associated with residents’ frailty levels but none of the facilities had < 60 residents. Another Australian research including smaller facilities suggests that service size might be associated with residents’ frailty status [46]. Ambagtsheer et al. [46] found a significant association between RACS size and frailty levels with bigger services (61–80 beds) having more frail residents compared to smaller services (1–60 beds).

### Strengths and limitations

A major strength of this study was the design methodology focused on making it possible for those with dementia to participate. To enable participation whilst reducing the survey burden, we had to include reconstructed scales, observations and medical records rather than exclusively relying on resident interviews. The mean recruitment rate of 55.5% in this study however was slightly lower compared to our previous research (63.5%), which included six of the twelve RACS involved in this study but the mean age (87.7 vs. 87.5 years) and proportion of females (73.3 vs. 77.5%) were similar [41]. The lower recruitment rate is explained in part by survey fatigue arising from internal surveys and accreditations as well as the increasing prevalence of dementia affecting the consent process. This research is confined to one of the largest residential aged care organizations in South Australia limiting somewhat the generalizability of the findings. For example, when compared to the 2015 findings of the Registry of Senior Australians (ROSA), a national registry of aged care administrative data, the mean age was similar (87.7 vs. 86.0 years), but this study had a greater proportion of females (73.3 vs. 60.9%) and the median FI, although using different variables, was higher (0.36 vs. 0.23) [6].

## Conclusion

This study suggests that RACS in Australia are increasingly being called upon to manage very frail older people, many of whom have geriatric syndromes such as dementia. In providing more appropriate response, staff will need to be better skilled in the management of geriatric syndromes including frailty and funding mechanisms will need to support the better management of these conditions in RACS if unnecessary upstream transfers to hospitals are to be avoided.

## Supplementary Information

**Additional file 1.** FIRST Study Data Collection Booklet.

**Additional file 2.** FIRST Study 60-Item Frailty Index.

**Additional file 3.** FIRST Study FRAIL-NH.

## Data Availability

The datasets generated and/or analysed as part of this longitudinal study are not publicly available because the team has multiple planned analysis yet to be conducted, and data collection and data linkage is still in process (anticipated to be completed by 2024). Beyond 2024, de-identified data may be made available following request from the chief investigator (RV) and will need to meet specified requirements that will be better defined by 2024. These requirements are likely to include (but not limited to) ethics approval from the University of Adelaide (a responsibility of the requestor), project approval by the aged care organisation, not overlapping with existing research projects, signing of a data use agreement, and cost recovery for data provision.

## Abbreviations

ATC: Anatomical Therapeutic Chemical
CCI-6D: Consumer Choice Index-6 Dimension
COPD: Chronic obstructive pulmonary disease
DHS: Department of Human Services
DSRS: Dementia Severity Rating Scale
ESS: Epworth Sleepiness Scale
EREC: External Request Evaluation Committee
FI: Frailty Index
FIRST: Frailty In Residential Sector over Time
GP: General Practitioner
HREC: Human Research Ethics Committee
ISAAC: Integrated South Australian Activity Collection
KATZ-ADL: Katz Activities of Daily Living
MBS: Medicare Benefits Schedule
MI: Myocardial Infarction
MNA-SF: Mini Nutritional Assessment Short Form
NHLSD: Nursing Home Life Space Diameter
PAINAD: Pain Assessment in Advanced Dementia
PAS: Psychogeriatric Assessment Scale
PBS: Pharmaceutical Benefits Scheme
PHQ-4: Patient Health Questionnaire-4
POSS: Pasero Opioid Induced Sedation Scale
PRN: Pro Re Nata
PWI: Personal Wellbeing Index
QoL-AD: Quality of Life Alzheimer Disease
RACS: Residential Aged Care Service
Redcap: Research Electronic Data Capture
ROC: Receiver operating characteristic
SARC-F: Screening Tool Sarcopenia
SDM: Substitute decision maker
SD: Standard Deviation
