# Supplementary material for: The Frailty In Residential Sector over Time (FIRST) study: methods and baseline cohort description
Source: BMC Geriatr. 2021 Feb 3;21:99. doi: 10.1186/s12877-020-01974-1 (PMC7857100; doi:10.1186/s12877-020-01974-1)
Supplement: Supplementary file 1 — Additional file 1. FIRST Study Data Collection Booklet. [file 12877_2020_1974_MOESM1_ESM.pdf]

Additional File 1:

DATA COLLECTION BOOKLET

Frailty In Residential Sector Over Time (FIRST) Study

Assessments

Study nurse to obtain resident’s consent:

1. Resident’s consent and time of data collection

Study nurse to complete using resident’s records:

2. Resident’s Information
3. Mini Nutritional Assessment Short Form (MNA-SF) (<3 months)
4. Katz Activities of Daily Living Scale (KATZ ADL)

Study nurse to upload resident’s records:

5. Resident’s Records

Study nurse to observe the resident:

6. Pasero Opioid Induced Sedation Scale (POSS)
7. Pain Assessment in Advanced Dementia Scale (PAINAID)

Study nurse to complete with resident (or else omit):

8. Grip Strength Assessment
9. Consumer Choice Index 6 Dimension (CCI-6D)
10. Personal Wellness Index (PWI)

Study nurse to complete with resident (or else with site registered nurse):

11. Quality of Life in Alzheimer’s Disease Scale (QOL-AD)
12. Epworth Sleepiness Scale (ESS)
13. Sleep Question
14. Patient Health Questionnaire-4 (PHQ-4)

Study nurse to complete with site registered nurse:

15. SARC-F
16. Nursing Home Life-Space Diameter (NHLSD)
17. Dementia Severity Rating Scale (DSRS)
18. Mini Nutritional Assessment Short Form (MNA-SF) (≥3 months)

Research staff to complete data entry:

19. Socio-Demographic Information
20. Co-Morbidities
21. Medication Use

1. CONSENT & TIME OF DATA COLLECTION

Study nurse to obtain resident's consent

1.1 Has resident's consent been obtained?

yes [ ]   no [ ]   by resident [ ] or substitute decision-maker [ ]   Date of consent __________

1.2 Time of data collection: baseline [ ] 1 year [ ]

2. RESIDENT'S INFORMATION

Study nurse to complete from resident's records

2.1. Number of falls over the last 12 months ___________

2.2. Weight: current _______kg   last month______ kg   2 months ago ______kg   3 months ago _____kg

2.3. Height ____cm → if not in records measure ulna _____cm (automatic calculate height from ulna cm)

2.4. Body Mass Index_____________ (automatically calculate from weight/height^2)

3. MINI NUTRITIONAL ASSESSMENT SHORT FORM (MNA-SF)

Study nurse to complete from resident's records

3.1. Mini Nutritional Assessment Short Form older than 3 months?

Yes [ ]   No [ ]   Date: _______

If No: Please answer the following questions using resident's MNA-SF records:

3.1.1. Has food intake declined over the past 3 months due to loss of appetite, digestive problems, chewing or swallowing difficulties?
[0] = severe decrease in food intake
[1] = moderate decrease in food intake
[2] = no decrease in food intake

3.1.2. Has suffered psychological stress or acute illness in the past 3 months?
[0] = Yes   [2] = No

Source: Guigoz Y, Lauque S, Vellas BJ. Identifying the elderly at risk for malnutrition. The Mini Nutritional Assessment. Clin Geriatr Med. 2002;18(4):737-57.

4. KATZ ACTIVITIES OF DAILY LIVING SCALE (KATZ ADL)

Study nurse to complete using resident's records/plans of care

Please mark 'yes' if the statement applies for the resident:

4.1. Bathing
The resident receives no assistance with their usual way of bathing   □ Yes[1]   □ No[0]

4.2. Dressing
The resident is able to get their clothes and get completely dressed without help   □ Yes[1]   □ No[0]

4.3. Toileting
The resident is able to go to the toilet, get on and off the toilet and attend to their personal hygiene on my own — □ Yes[1] □ No[0]

4.4. Transferring
The resident is able to move in and out of bed as well as get on and off chairs on their own. In doing this, he/she might be using a stick or walker etc. for assistance but he/she doesn't need help from other people. — □ Yes[1] □ No[0]

4.5. Continence — □ Yes[1] □ No[0]
The resident manages his/her bladder and bowels independently and has no 'accidents'. He/she may be using an incontinence pad to avoid 'accidents'

4.6. Feeding — □ Yes[1] □ No[0]
The resident is able to feed himself/herself from the plate on their own. This includes cutting food, buttering bread etc.The food might be prepared by someone else.

Total Score: _____ / 6

Source: Shelkey M, Wallace M. Katz Index of Independence in Activities of Daily Living (ADL). Director. 2000;8(2):72-3.

5. UPLOAD RESIDENTS' RECORDS

Study nurse to upload resident's information:

5.1. Upload Socio-Demographic Information
5.2. Upload Medical Health History
5.3. Upload Medication Chart

6. PASERO OPIOD INDUCED SEDATION SCALE (POSS)

Study nurse to observe the resident

Instructions: Please indicate after observing for a period of 5 minutes rest if the resident is:

6.1. [S] Sleep, easy to arouse
Acceptable; no action necessary; may increase opioid dose if needed

6.2. [1] Awake and alert
Acceptable; no action necessary; may increase opioid dose if needed

6.3. [2] Slightly drowsy, easily aroused
Acceptable; no action necessary; may increase opioid dose if needed

6.4. [3] Frequently drowsy, arousable, drifts off to sleep during conversation
Unacceptable; monitor respiratory status and sedation level closely until sedation level is stable at less than 3 and respiratory status is satisfactory; decrease opioid dose 25% to 50% or notify prescriber or anaesthesiologist for orders; consider administering a non-sedating, opioid-sparing nonopioid, such as acetaminophen or a NSAID, if not contraindicated.

6.5. [4] Somnolent, minimal or no response to verbal and physical stimulation
Unacceptable; stop opioid; consider administering naloxone; notify prescriber or anaesthesiologist; monitor respiratory status and sedation level closely until sedation level is stable at less than 3 and respiratory status is satisfactory.

Source: Pasero C, McCaffery M. Monitoring sedation. Am J Nurs. 2002;102(2):67-9.

7. PAIN ASSESSMENT IN ADVANCED DEMENTIA SCALE (PAINAID)

Study nurse to observe the resident

Instructions: Observe the resident for 5 minutes before scoring his or her behaviours.

Please score the behaviours according to the following chart.

Definitions of each item are available **here** (link to more information). The resident can be observed under different conditions (e.g., at rest, during a pleasant activity, during caregiving, after the administration of pain medication).

Behaviour	0	1	2	Score
7.1. Breathing<br>Independent of<br>vocalization	Normal	Occasional labored breathing<br><br>Short period of hyperventilation	Noisy labored breathing<br>Long period of hyperventilation<br>Cheyne-Stokes respirations	
7.2. Negative vocalization	None	Occasional moan or groan<br><br>Low-level speech with a negative or	Repeated troubled calling out<br>Loud moaning or groaning<br>Crying	
7.3. Facial expression	Smiling or inexpressive	Sad<br>Frightened<br>Frown	Facial grimacing	
7.4. Body language	Relaxed	Tense<br>Distressed pacing<br>Fidgeting	Rigid<br>Fists clenched<br>Knees pulled up<br>Pulling or pushing away<br>Striking out	
7.5. Consolability	No need to console	Distracted or reassured by voice or touch	Unable to console, distract, or reassure	

Total Score: ___ / 10

[ ] 1-3=mild pain [ ] 4-6=moderate pain [ ] 7-10=severe pain

Source: Warden V, Hurley AC, Volicer L. Development and psychometric evaluation of the Pain Assessment in Advanced Dementia (PAINAD) scale. J Am Med Dir Assoc. 2003;4(1):9-15.

8. GRIP STRENGTH ASSESSMENT

Study nurse to complete with the resident

Instructions: The ball rests in the resident's hollow hand with its largest volume and the hose on the neck of the ball protrudes between thumb and index finger. The fingers that surround the ball should be quickly closed to a fist and by doing so should press the ball together. The finger tips should also be involved when closing the fingers to a fist that means they should firmly press into the ball.

8.1. Martin-Vigorimeter: Dominant Hand: [ ] right [ ] left Strength: [0] 0.7-1.3 bar [1] <0.69 bar [2] unable

9. CONSUMER CHOICE INDEX 6 DIMENSION (CCI-6D)

Study nurse to complete with the resident (only where possible or else omit)

Instructions:
Please answer these questions on how you feel about the care you are receiving in this facility over the past few weeks. (Please tick one option from the 6 questions below)

9.1. How much time are caregiving staff able to spend with you?
[3] Care staff are always able to spend enough time attending to my individual needs
[2] Care staff are sometimes able to spend enough time attending to my individual needs
[1] Care staff are rarely able to spend enough time attending to my individual needs

9.2. Do the shared spaces of the aged care home as a whole make you feel 'at home'?
[3] I feel very at home here
[2] I feel at home here sometimes
[1] I feel at home here rarely

9.3. Does your own room here make you feel 'at home'?
[3] I feel very at home in my room
[2] I feel at home in my room sometimes
[1] I feel at home in my room rarely

9.4. Is there access to outside and gardens in this aged care home?
[3] I can outside whenever I want
[2] I can get outside sometimes
[1] I cannot get outside easily

9.5. How often does the facility offer you things to do that make you feel valued?
[3] I can do things that make me feel valued often
[2] I can sometimes do things that make me feel valued
[1] I can only rarely or occasionally do things that make me feel valued

9.6. How flexible is the aged care home with the care routines (e.g. when you get out of bed, shower, eat your meals)
[3] Care routines are very flexible. The aged care home is very happy to change the times they provide help if I require it
[2] There is a little flexibility in the care routines. The aged care home sometimes changes the times they provide care if I ask for it
[1] There is not much flexibility in the care routines. Care and assistance seems to occur when it most suits the aged care home

Source: Milte R, Ratcliffe J, Bradley C, Shulver W, Crotty M. Evaluating the quality of care received in long-term care facilities from a consumer perspective: development and construct validity of the Consumer Choice Index Six Dimension instrument. Ageing Soc. 2019; 39(1):138-60

10. PERSONAL WELLNESS INDEX (PWI)

Study nurse to complete with the resident (only where possible or else omit)

Instructions:
I am now going to ask how satisfied you feel, on a scale from zero to 10. Zero means you feel no satisfaction at all. 10 means you feel completely satisfied.

No satisfaction at all										Completely satisfied
0	1	2	3	4	5	6	7	8	9	10

Would you like me to go over this again for you? [If "yes", repeat the above. If "no", proceed to next statement] In that case, I will start.

10.1. Part I (optional]: Satisfaction with Life as a Whole

Thinking about your own life and personal circumstances, how satisfied are you with your life as a whole? Score ____

10.2. Part II: Personal Wellbeing Index

How satisfied are you with…		
	1. your standard of living ?	Score ____
	2. your health ?	Score ____
	3. what you are achieving in life ?	Score ____
	4. your personal relationships ?	Score ____
	5. how safe you feel ?	Score ____
	6. feeling part of your community ?	Score ____
	7. your future security ?	Score ____
	8. your spirituality or religion? [optional]	Score ____

Source: International Wellbeing Group (2013). Personal Wellbeing Index: 5th Edition. Melbourne: Australian Centre on Quality of Life, Deakin University.

11. QUALITY OF LIFE IN ALZHEIMER'S DISEASE SCALE (QOL-AD)

Study nurse to complete with the resident (if resident unable, to complete with site registered nurse)

The Quality of Life in Alzheimer's Disease (QOL-AD) questionnaire is copyright protected.
Contact information and permission to use obtained from Mapi Research Trust, Lyon, France - Internet: https://eprovide.mapi-trust.org

12. EPWORTH SLEEPINESS SCALE (ESS)

Study nurse to complete with the resident (if resident unable, to complete with site registered nurse)

The Epworth Sleepiness Scale (ESS) is copyright protected.
Contact information and permission to use obtained from Mapi Research Trust, Lyon, France - Internet: https://eprovide.mapi-trust.org

13. SLEEP QUESTION

Study nurse to complete with the resident (if resident unable, to complete with site registered nurse)

Completed by [ ] resident or [ ] site registered nurse

13.1. How regularly do(es) you (the resident) sleep, nap or lie down during the week?

[1] nearly every day
[2] a few times a week
[3] once or twice a week
[4] once or twice a month
[5] never or hardly ever

Source: St George RJ, Delbaere K, Williams P, Lord SR. Sleep quality and falls in older people living in self- and assisted-care villages. Gerontology. 2009;55(2):162-8.

14. PATIENT HEALTH QUESTIONNAIRE-4 (PHQ-4)

Study nurse to complete with the resident (if resident unable, to complete with site registered nurse)

Completed by [ ] resident or [ ] site registered nurse

Over the last two weeks, how often have (has) you (the resident) been bothered by the following problems?

14.1. Feeling nervous, anxious or on edge
[0] Not at all
[1] Several days
[2] More than half the days
[3] Nearly every day

14.2. Not being able to stop or control worrying
[0] Not at all
[1] Several days
[2] More than half the days
[3] Nearly every day

Total score ≥3 for first 2 questions suggests anxiety: Score ____

14.3. Feeling down, depressed or hopeless
[0] Not at all
[1] Several days
[2] More than half the days
[3] Nearly every day

14.4. Little interest or pleasure in doing things
[0] Not at all
[1] Several days
[2] More than half the days
[3] Nearly every day

Total score ≥3 for last 2 questions suggests depression: Score _____

Total Score: _____ / 12

[ ] normal (0-2) [ ] mild (3-5) [ ] moderate (6-8) [ ] severe (9-12)

Source: Kroenke K, Spitzer RL, Williams JB, Lowe B. An ultra-brief screening scale for anxiety and depression: the PHQ-4. Psychosomatics. 2009;50(6):613-21.

15. SARC-F

Study nurse to complete with site registered nurse

15.1. Strength	[0] 0.7 - 1.3 bar [1] <0.69 bar [2] unable (prepopulated from 8.1)<br>dominant hand [ ] right [ ] left
15.2. Assistance with walking	How much difficulty does the resident have walking across a room?<br>[0] None [1] Some [2] A lot, use aids or unable
15.3. Rise from chair	How much difficulty does the resident have transferring from a chair or bed?<br>[0] None [1] Some [2] A lot or unable without help
15.4. Climb stairs	How much difficulty does the resident have climbing a flight of 10 stairs?<br>[0] None [1] Some [2] A lot or unable
15.5. Falls	How many times has the resident fallen in the past 12 months?<br>[0] None [1] 1-3 falls [2] ≥ 4 falls

Total Score ______ / 10
(≥ 4: at-risk of sarcopenia)

Source: Woo J, Leung J, Morley JE. Validating the SARC-F: a suitable community screening tool for sarcopenia? J Am Med Dir Assoc. 2014;15(9):630-4.

16. NURSING HOME LIFE-SPACE DIAMETER (NHLSD)

Study nurse to complete with site registered nurse

Within the last 2 weeks, how often has the resident moved around:

Diameter	Frequency
16.1 Within his or her own room	[0] Never<br>[1] Less than weekly<br>[2] At least weekly<br>[3] >2 times a week<br>[4] 1-3 times a day<br>[5] >3 times a day
16.2 Outside the room, within the unit	[0] Never<br>[1] Less than weekly<br>[2] At least weekly<br>[3] >2 times a week<br>[4] 1-3 times a day<br>[5] >3 times a day

16.3 Outside the unit, throughout the facility
[0] Never
[1] Less than weekly
[2] At least weekly
[3] >2 times a week
[4] 1-3 times a day
[5] >3 times a day

16.4 Outside the facility (i.e., left the facility)
[0] Never
[1] Less than weekly
[2] At least weekly
[3] >2 times a week
[4] 1-3 times a day
[5] >3 times a day

Source: Tinetti ME, Ginter SF. The nursing home life-space diameter. A measure of extent and frequency of mobility among nursing home residents. J Am Geriatr Soc. 1990;38(12):1311-5.

17. DEMENTIA SEVERITY RATING SCALE (DSRS)

Study nurse to complete with site registered nurse

How much contact do you have with the resident?
[1] Less than 1 day per week
[2] 1day/week
[3] 2 days/week
[4] 3-4 days/week
[5] 5 or more days per week

In each section, please circle the number that most closely applies to the resident. This is a general form, so no one description may be exactly right, please circle the answer that seems to apply most of the time.

Please circle only one number per section, and be sure to answer all questions.

17.1. MEMORY
[0] Normal memory
[1] Occasionally forgets things that they were told recently. Does not cause many problems
[2] Mild consistent forgetfulness. Remembers recent events but often forgets parts
[3] Moderate memory loss. Worse for recent events. May not remember something you just told them. Causes problems with everyday activities
[4] Substantial memory loss. Quickly forgets recent or newly-learned things. Can only remember things that they have known for a long time
[5] Does not remember basic facts like the day of the week, when last meal was eaten or what the next meal will be
[6] Does not remember even the most basic things

17.2. SPEECH AND LANGUAGE
[0] Normal ability to talk and to understand others
[1] Sometimes cannot find a word, but able to carry on conversations
[2] Often forgets words. May use the wrong word in its place. Some trouble expressing thoughts and giving answers

[3] Usually answers questions using sentences but rarely starts a conversation
[4] Answers questions, but responses are often hard to understand or don't make sense. Usually able to follow simple instructions
[5] Speech often does not make sense. Cannot answer questions or follow instructions
[6] Does not respond most of the time

17.3. RECOGNITION OF FAMILY MEMBERS
[0] Normal - recognizes people and generally knows who they are.
[1] Usually recognizes grandchildren, cousins or relatives who are not seen frequently but may not recall how they are related.
[2] Usually does not recognize family members who are not seen frequently. Is often confused about how family members such as grandchildren, nieces, or nephews are related to them.
[3] Sometimes does not recognize close family members or others who they see frequently. May not recognize their children, brothers, or sisters who are not seen on a regular basis.
[4] Frequently does not recognize spouse or caregiver.
[5] No recognition or awareness of the presence of others.

17.4. ORIENTATION TO TIME
[0] Normal awareness of time of day and day of week.
[1] Some confusion about what time it is or what day of the week, but not severe enough to interfere with everyday activities.
[2] Frequently confused about time of day.
[3] Almost always confused about the time of day.
[4] Seems completely unaware of time.

17.5. ORIENTATION TO PLACE
[0] Normal awareness of where they are even in new places.
[1] Sometimes disoriented in new places.
[2] Frequently disoriented in new places.
[3] Usually disoriented, even in familiar places. May forget that they are already at home.
[4] Almost always confused about place.

17.6. ABILITY TO MAKE DECISIONS
[0] Normal - as able to make decisions as before.
[1] Only some difficulty making decisions that arise in day-to-day life.
[2] Moderate difficulty. Gets confused when things get complicated or plans change.
[3] Rarely makes any important decisions. Gets confused easily.
[4] Not able to understand what is happening most of the time.

17.7. SOCIAL AND COMMUNITY ACTIVITY
[0] Normal - acts the same with people as before
[1] Only mild problems that are not really important, but clearly acts differently from previous years.
[2] Can still take part in community activities without help. May appear normal to people who don't know them.
[3] Often has trouble dealing with people outside the home without help from caregiver. Usually can participate in quiet home activities with friends. The problem is clear to anyone who sees them.
[4] No longer takes part in any real way in activities at home involving other people. Can only deal with the primary caregiver.
[5] Little or no response even to primary caregiver.

17.8. HOME ACTIVITIES AND RESPONSIBILITIES

[0] Normal. No decline in ability to do things around the house.
[1] Some problems with home activities. May have more trouble with money management (paying bills) and fixing things. Can still go to a store, cook or clean. Still watches TV or reads a newspaper with interest and understanding.
[2] Makes mistakes with easy tasks like going to a store, cooking or cleaning. Losing interest in the newspaper, TV or radio. Often can't follow a long conversation on a single topic.
[3] Not able to shop, cook or clean without a lot of help. Does not understand the newspaper or the TV. Cannot follow a conversation.
[4] No longer does any home-based activities.

17.9. PERSONAL CARE - CLEANLINESS

[0] Normal. Takes care of self as well as they used to.
[1] Sometimes forgets to wash, shave, comb hair, or may dress in wrong type of clothes. Not as neat as they used to be.
[2] Requires help with dressing, washing and personal grooming.
[3] Totally dependent on help for personal care.

17.10. EATING

[0] Normal, does not need help in eating food that is served to them.
[1] May need help cutting food or have trouble with some foods, but basically able to eat by themselves.
[2] Generally able to feed themselves but may require some help. May lose interest during the meal.
[3] Needs to be fed. May have trouble swallowing.

17.11. CONTROL OF URINATION AND BOWELS

[0] Normal - does not have problems controlling urination or bowels except for physical problems.
[1] Rarely fails to control urination (generally less than one accident per month).
[2] Occasional failure to control urination (about once a week or less).
[3] Frequently fails to control urination (more than once a week).
[4] Generally fails to control urination and frequently can not control bowels.

17.12. ABILITY TO GET FROM PLACE TO PLACE

[0] Normal, able to get around on their own. (May have physical problems that require a cane or walker).
[1] Sometimes gets confused when driving or taking public transportation, especially in new places. Able to walk places alone.
[2] Cannot drive or take public transportation alone, even in familiar places. Can walk alone outside for short distances. Might get lost if walking too far from home.
[3] Cannot be left outside alone. Can get around the house without getting lost or confused.
[4] Gets confused and needs help finding their way around the house.
[5] Almost always in a bed or chair. May be able to walk a few steps with help, but lacks sense of direction.
[6] Always in bed. Unable to sit or stand.

Total Score: _____ / 54

[ ] 0-18 Mild [ ] 19-36 Moderate [ ] 37-54 Severe

Source: Clark CM, Ewbank DC. Performance of the dementia severity rating scale: a caregiver questionnaire for rating severity in Alzheimer disease. Alzheimer Dis Assoc Disord. 1996;10(1):31-9.

18. MINI NUTRITIONAL ASSESSMENT SHORT FORM (MNA-SF)

Study nurse to complete with site registered nurse

Date ______ /_______ / _______ (may be prepopulated from 3.1)
Weight (kg, one decimal point): ________kg (prepopulated from 2.2)
Height (m, two decimal points): ________m (prepopulated from 2.3)
Body mass index (weight, kg/height^2, m^2): _____________ (prepopulated from 2.4)

18.1. Has food intake declined over the past 3 months due to loss of appetite, digestive problems, chewing or swallowing difficulties? (may be prepopulated from 3.1.1 if MNA-SF <3 months old)
[0] = severe decrease in food intake
[1] = moderate decrease in food intake
[2] = no decrease in food intake

18.2. Weight loss during the last 3 months (prepopulated from 2.2)
[0] = weight loss >3 kg [or response is ‘a lot’]
[1] = does not know
[2] = weight loss between 1 and 3 kg [or response is ‘a little’]
[3] = no weight loss

18.3. Mobility
[0] = bed or chair bound (prepopulated from 17.12 score 0-2)
[1] = able to get out of bed/chair but does not go out (prepopulated from 17.12 score 3-4)
[2] = goes out (prepopulated from 17.12 score 5-6)

18.4. Has suffered psychological stress or acute illness in the past 3 months? (may be prepopulated from 3.1.2 if MNA-SF >3 months old)
[0] = Yes
[2] = No

18.5. Neuropsychological problems
[0] = severe dementia or depression (prepopulated from 17 total score ≥19 and 14.3+14.4 is ≥3)
[1] = mild dementia (prepopulated from 17 total score 12-18)
[2] = no psychological problems (prepopulated from 17 total score <12 and 14.3+14.4 is <3)

18.6. Body mass index (BMI) (prepopulated from 2.4)
[0] = BMI <19
[1] = BMI 19-<21
[2] = BMI 21-<23
[3] = BMI 23/<

Total Score: _____ / 14

Source: Guigoz Y, Lauque S, Vellas BJ. Identifying the elderly at risk for malnutrition. The Mini Nutritional Assessment. Clin Geriatr Med. 2002;18(4):737-57.

19. SOCIO-DEMOGRAPHIC INFORMATION

Research staff to complete from uploads (resident's records)

Resthaven Inc. Aged Care Facility Postcode:

[1] Craigmore SA 5114	[5] Mitcham SA 5062	[9] Port Elliot SA 5212
[2] Leabrook SA 5068	[6] Mount Gambier SA 5290	[10] Westbourne Park SA 5041
[3] Malvern SA 5061	[7] Murray Bridge SA 5253	[11] Bellevue Heights SA 5050
[4] Marion SA 5043	[8] Paradise SA 5075	

Resident Name:

First name ________________ Middle Name:_______________ Surname:_________________

Medicare Number: __________________________

Gender: [1] Male [2] Female DOB: _____/______/______

Primary Language: [1] English [2] Not English

Country of Birth: ________________________

20. CO-MORBIDITIES

Research staff to complete from uploads (resident's records)

Myocardial infarction*	[1] Yes	[0] No
Congestive cardiac failure*	[1] Yes	[0] No
Peripheral vascular disease*	[1] Yes	[0] No
Cerebrovascular disease (or stroke)*	[1] Yes	[0] No
Atrial fibrillation*	[1] Yes	[0] No
Hypertension*	[1] Yes	[0] No
Diabetes	[1] Yes	[0] No
History of Delirium	[1] Yes	[0] No
Parkinson's Disease	[1] Yes	[0] No
Dementia*	[1] Yes	[0] No
Depression*	[1] Yes	[0] No
Anxiety	[1] Yes	[0] No
Chronic obstructive pulmonary disease/Asthma*	[1] Yes	[0] No
Arthritis*	[1] Yes	[0] No
Osteoporosis*	[1] Yes	[0] No
Hip fracture*	[1] Yes	[0] No
Other fractures*	[1] Yes, please specify	[0] No

Femur (thigh bone)	[ ]
Pelvis	[ ]
Vertebrae (spine)	[ ]
Upper arm / Forearm / Hand	[ ]
Leg	[ ]
Ankles	[ ]
Other	[ ]

Gout*	[1] Yes	[0] No
Pressure sores*	[1] Yes	[0] No
Leg ulcers	[1] Yes	[0] No
Ulcer disease*	[1] Yes	[0] No
Connective tissue disease*	[1] Yes	[0] No
Urinary incontinence*	[1] Yes	[0] No
Faecal incontinence	[1] Yes	[0] No
Skin cancers	[1] Yes	[0] No
Other cancers	[1] Yes, please specify	[0] No

- Breast cancer [ ]
- Prostate cancer [ ]
- Colon cancer [ ]
- Lung cancer [ ]
- Stomach/gastric cancer [ ]
- Other [ ]

Any tumor*	[1] Yes	[0] No
Hearing Impairment	[1] Yes	[0] No
Dry eyes*	[1] Yes	[0] No
Glaucoma	[1] Yes	[0] No
Insomnia* (prepopulated from 12, yes if total score ≥11)	[1] Yes	[0] No
Falls* (prepopulated from 2.1)	[1] Yes	[0] No

*22 Comorbidities relevant for the Frailty Index

21. MEDICATION USE

Research staff to complete from uploads (resident's records)

Name	Strength	Doses	Administered yes/no	ATC classification

Polypharmacy ≥ 9 [1] Yes [0] No
