# Supplementary material for: The Frailty In Residential Sector over Time (FIRST) study: methods and baseline cohort description
Source: BMC Geriatr. 2021 Feb 3;21:99. doi: 10.1186/s12877-020-01974-1 (PMC7857100; doi:10.1186/s12877-020-01974-1)
Supplement: Supplementary file 2 — Additional file 2. FIRST Study 60-Item Frailty Index. [file 12877_2020_1974_MOESM2_ESM.pdf]

Additional File 2: FIRST Study 60-Item Frailty Index

60-Item Frailty Index (FI)		
Myocardial infarction	Any tumor	Dozing while watching TV[e]
Congestive heart failure	Falls	Dozing while sitting in public[e]
Peripheral vascular disease	Bathing[a]	Dozing as a passenger in a car[e]
Cerebrovascular disease	Dressing[a]	Dozing while lying down to rest[e]
Dementia	Toileting[a]	Dozing while sitting after lunch[e]
Chronic pulmonary disease	Physical health[b]	Napping frequency[f]
Connective tissue disease	Energy[b]	Facial expression[g]
Ulcer disease	Mood[b]	Body language[g]
Hypertension	Living situation[b]	Memory[h]
Atrial fibrillation	Family[b]	Speech and language[h]
Insomnia	Friends[b]	Recognition of family members[h]
Depression	Self as a whole[b]	Orientation to time[h]
Arthritis	Ability to do things for fun[b]	Orientation to place[h]
Hip fracture	Life as a whole[b]	Ability to make decisions[h]
Other fracture	Food intake decline[c]	Social and community activity[h]
Osteoporosis	Weight loss[c]	Home activities and responsibilities[h]
Gout	Neuropsychological problems[c]	Personal care, cleanliness[h]
Pressure sores	Body mass index, calf circumference[c]	Eating[h]
Dry eyes	Sedation scale[d]	Control of urination and bowels[h]
Urinary incontinence	Dozing while reading[e]	Ability to get from place to place[h]

FI Categories: non-frail (0 to ≤0.1), vulnerable (>0.1 to 0.21), frail (>0.21 to <0.45), most-frail (0.45 or more); [a]Katz Activities of Daily Living Scale, [b]13-Item Quality of Life in Alzheimer's Disease Scale, [c]Mini Nutritional Assessment Short Form, [d]Pasero Opioid Induced Sedation Scale, [e]Epworth Sleepiness Scale, [f]Sleep Quality Questionnaire, [g]Pain Assessment in Advanced Dementia Scale, [h]Dementia Severity Rating Scale
