# Supplementary material for: The Frailty In Residential Sector over Time (FIRST) study: methods and baseline cohort description
Source: BMC Geriatr. 2021 Feb 3;21:99. doi: 10.1186/s12877-020-01974-1 (PMC7857100; doi:10.1186/s12877-020-01974-1)
Supplement: Supplementary file 3 — Additional file 3. FIRST Study FRAIL-NH. [file 12877_2020_1974_MOESM3_ESM.pdf]

Additional File 3: FIRST Study FRAIL-NH

**Variable**	**0**	**1**	**2**
Energy[a]	Feeling down, depressed or hopeless: not at all (0)	Feeling down, depressed or hopeless: several days (1) **OR** more than half the days (2)	Feeling down, depressed or hopeless **AND** Little interest or pleasure (total ≥3)
Transferring[b]	Difficulty transferring from chair/bed: none (0)	Difficulty transferring from chair/bed: Some (1)	Difficulty transferring from chair/bed: A lot/unable without help (2)
Mobility[c]	Goes out (DSRS #12 Score 0-2)	Able to get out of bed/chair but not go out (DSRS #12 Score 3-4)	Bed or chair bound (DSRS #12 Score 5-6)
Continence[c]	Normal **OR** rarely fails (DSRS #11 Score 0-1)	Occasionally **OR** frequently fails (DSRS #11 Score 2-3)	Generally fails (DSRS #11 Score 4)
Weight Loss[d]	No weight loss	1–3 kg **OR** does not know	>3 kg (6.6 pounds)
Feeding[c]	Normal (DSRS #10 Score 0)	May require help (DSRS #10 Score 1-2)	Needs to be fed (DSRS #10 Score 3)
Dressing[c]	Independent (DSRS #9 Score 0)	May require help (DSRS #9 Score 1-2)	Totally dependent (DSRS #9 Score 3)

Total score: 0-14, Categories: 0-2 non-frail, 3-6 frail, 7-14 most-frail
[a] Patient Health Questionnaire-4**;** [b] SARC-F**;** [c] Dementia Severity Rating Scale; [d] Mini Nutritional Assessment Short Form
